# Supplementary material for: Gene regulatory co-option drives birdsong neural circuit specialization
Source: bioRxiv. 2026 Apr 24:2026.04.24.720702. Preprint. [Version 1] doi: 10.64898/2026.04.24.720702 (PMC13131659; doi:10.64898/2026.04.24.720702)
Supplement: Supplement 1 [file NIHPP2026.04.24.720702v1-supplement-1.pdf]

bioRxiv preprint doi: https://doi.org/10.64898/2026.04.24.720702; this version posted April 24, 2026. The copyright holder for this preprint (which was not certified by peer review) is the author/funder, who has granted bioRxiv a license to display the preprint in perpetuity. It is made available under aCC-BY 4.0 International license.

Supplementary Information

Supplementary Discussion

Cell type labels compared to Colquitt, Merullo et al. 2021.

Each nidopallial song region contained two projection neuron types (Glut-HVC-1, Glut-HVC-2, Glut-LMAN-1, and Glut-LMAN-2), while the arcopallial song region RA contained a single projection neuron type (Glut-RA). This is fewer than the number of HVC and RA glutamatergic neuron types described in our previous work [20]. We attribute this difference to two factors. First, this dataset consists of only a single species and data type (the previous dataset contained nuclei from zebra finches and cells from Bengalese finches), reducing batch effects that may have generated additional clusters. Second, the inclusion of surrounding tissue here allowed us to disambiguate whether clusters found in song regions are *bona fide* regional clusters or are the result of dissection error.

In HVC, several cell type label changes are worth specifically mentioning. Glut-HVC-2 here is identical to HVC-Glut-3 in [20] (**Extended Data Fig. 2c**), corresponding to HVC_X projection neurons. In addition, Glut-NC-1 and Glut-NC-2 are similar to HVC-Glut-2 in the previous analysis (**Extended Data Fig. 2c**). Although these types appear to be present in HVC (**Fig. 1f**), they are not specific to HVC and received the more general ”NC” label. Similarly, HVC-Glut-5 in the 2021 analysis is labeled Glut-NC-3 here, given its much higher abundance in NC compared to HVC. Glut-HVC-1a lies intermediate to Glut-HVC-1 and Glut-NC-1 (**Fig. 1d**) and may represent cells transiting from the NC-1 state to the mature HVC-1 state.

LMAN projections

Like HVC, LMAN projects to both RA and Area X, yet current evidence indicates that these projections arise from a single projection population [71–73], leaving the projection identities of the two Glut-LMAN neuron types ambiguous. We propose that Glut-LMAN-2, which is transcriptionally similar to Glut-HVC-2 (**Fig. 2a**), is an uncharacterized population projecting to Area X (**Fig. 2b**).

bioRxiv preprint doi: https://doi.org/10.64898/2026.04.24.720702; this version posted April 24, 2026. The copyright holder for this preprint (which was not certified by peer review) is the author/funder, who has granted bioRxiv a license to display the preprint in perpetuity. It is made available under aCC-BY 4.0 International license.

Supplementary Figures

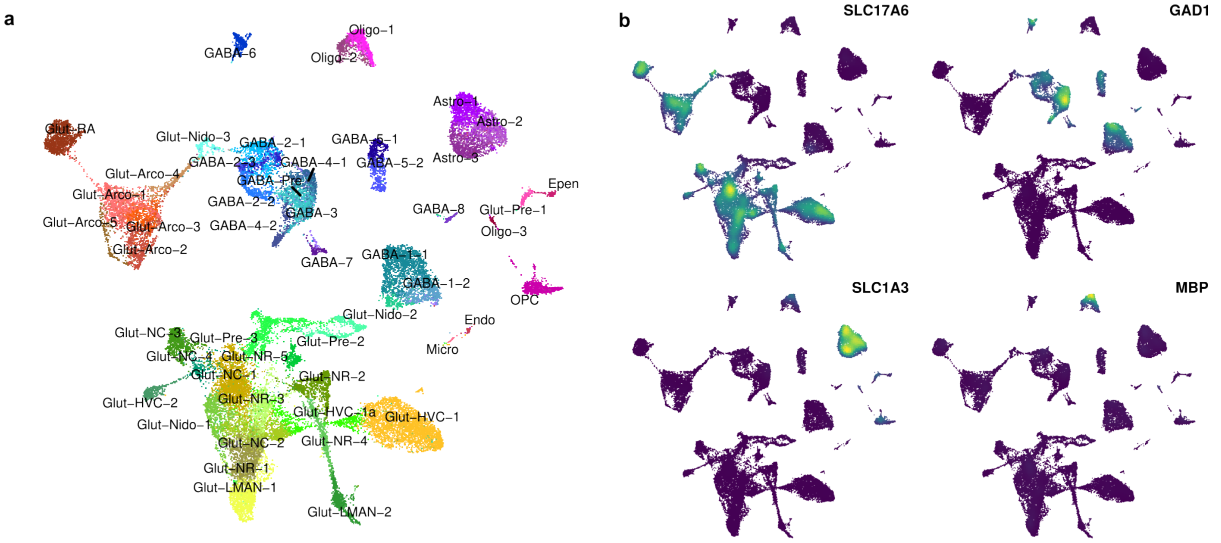

Extended Data Figure 1: Overview of cell types across song and non-song regions

(a) UMAP plot of the snRNA-seq dataset showing all clusters.
(b) UMAP plots showing the expression of *SLC17A6* (glutamatergic neurons), *GAD1* (GABAergic neurons), *SLC3A1* (astrocytes), and *MBP* (oligodendrocytes).

bioRxiv preprint doi: https://doi.org/10.64898/2026.04.24.720702; this version posted April 24, 2026. The copyright holder for this preprint (which was not certified by peer review) is the author/funder, who has granted bioRxiv a license to display the preprint in perpetuity. It is made available under aCC-BY 4.0 International license.

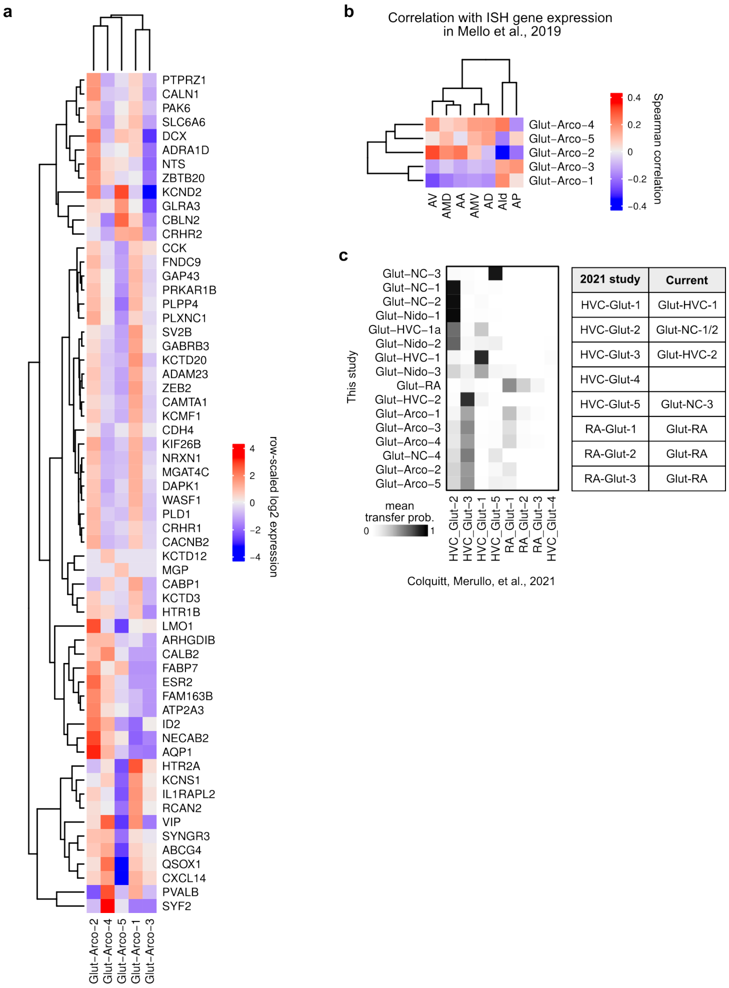

Extended Data Figure 2: Transcriptional similarities of arcopallial glutamatergic neurons to arcopallial subregions.

(a) Expression of the genes tested by *in situ* hybridization in [74] across the arcopallial glutamatergic populations described here.
(b) Pairwise Spearman correlations of gene expression between arcopallial glutamatergic neuron types (snRNA-seq) and arcopallial regions (ISH).
(c) Label transfer from [20] glutamatergic neuron labels to current glutamatergic neuron labels. (*Left*) Mean transfer probabilities from Seurat reciprocal PCA anchor-based integration. (*Right*) Table of label correspondences between the two studies.

bioRxiv preprint doi: https://doi.org/10.64898/2026.04.24.720702; this version posted April 24, 2026. The copyright holder for this preprint (which was not certified by peer review) is the author/funder, who has granted bioRxiv a license to display the preprint in perpetuity. It is made available under aCC-BY 4.0 International license.

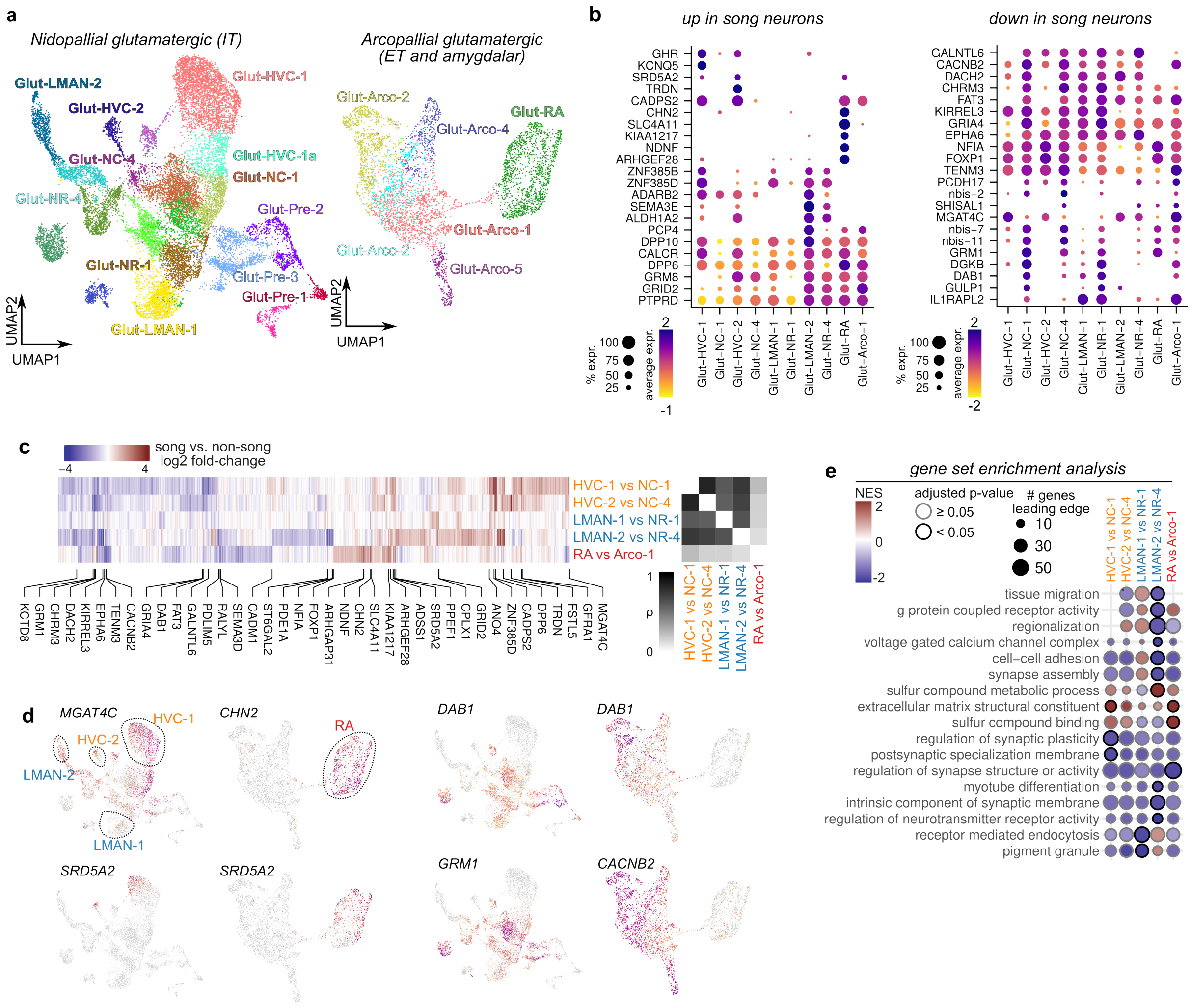

Extended Data Figure 3: Molecular specialization of song glutamatergic neurons

(a) UMAP plot of glutamatergic neuron clusters from the (*left*) nidopallium, including HVC and LMAN, and (*right*) arcopallium, including RA.
(b) Expression of the top five differentially expressed genes (up or down) for each song vs non-song pair.
(c) Heatmap of log2 fold changes in expression between each song glutamatergic neuron and its non-song counterpart. Grayscale heatmap shows the pairwise Spearman correlation of differential expression across each song neuron.
(d) UMAP of nidopallial and arcopallial glutamatergic cells showing the expression of example genes that elevated or reduced expression in song neurons.
(e) Gene set enrichment analysis of differentially expressed genes between each song and non-song glutamatergic neuron pair.

bioRxiv preprint doi: https://doi.org/10.64898/2026.04.24.720702; this version posted April 24, 2026. The copyright holder for this preprint (which was not certified by peer review) is the author/funder, who has granted bioRxiv a license to display the preprint in perpetuity. It is made available under aCC-BY 4.0 International license.

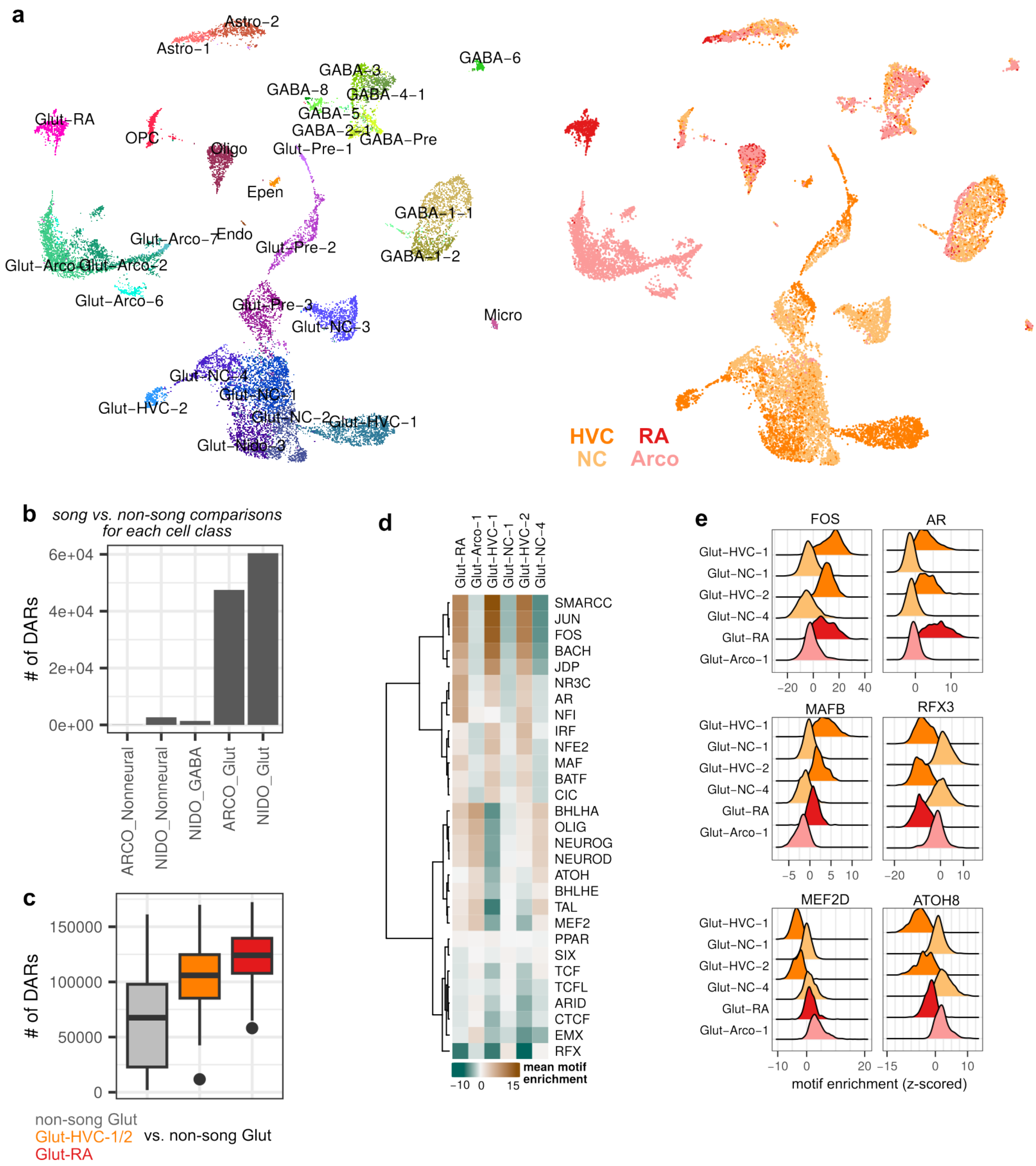

Extended Data Figure 4: Enrichment of transcription factor motifs in song versus non-song glutamatergic neurons

(a) UMAP plot of the Multiome dataset, expanded to show (*left*) all cluster labels and (*right*) dissection regions
(b) The number of differentially accessible regions (DARs) between song and non-song regions for each cell class.
(c) Distributions of the numbers of DARs across pairs of glutamatergic neuron types. Comparisons are divided into three sets: (1) non-song Glut neurons versus non-song Glut neurons, (2) Glut-HVC1/2 versus nidopallial non-song Glut neurons, and (3) Glut-RA versus arcopallial non-song Glut neurons.
(d) Mean enrichment of transcription factor binding site motifs across differentially accessible regions (DARs) in song and non-song glutamatergic types. Motifs are grouped by transcription factor family.
(e) Selected motifs that are commonly enriched or depleted across the DARs of each song glutamatergic neuron type.

bioRxiv preprint doi: https://doi.org/10.64898/2026.04.24.720702; this version posted April 24, 2026. The copyright holder for this preprint (which was not certified by peer review) is the author/funder, who has granted bioRxiv a license to display the preprint in perpetuity. It is made available under aCC-BY 4.0 International license.

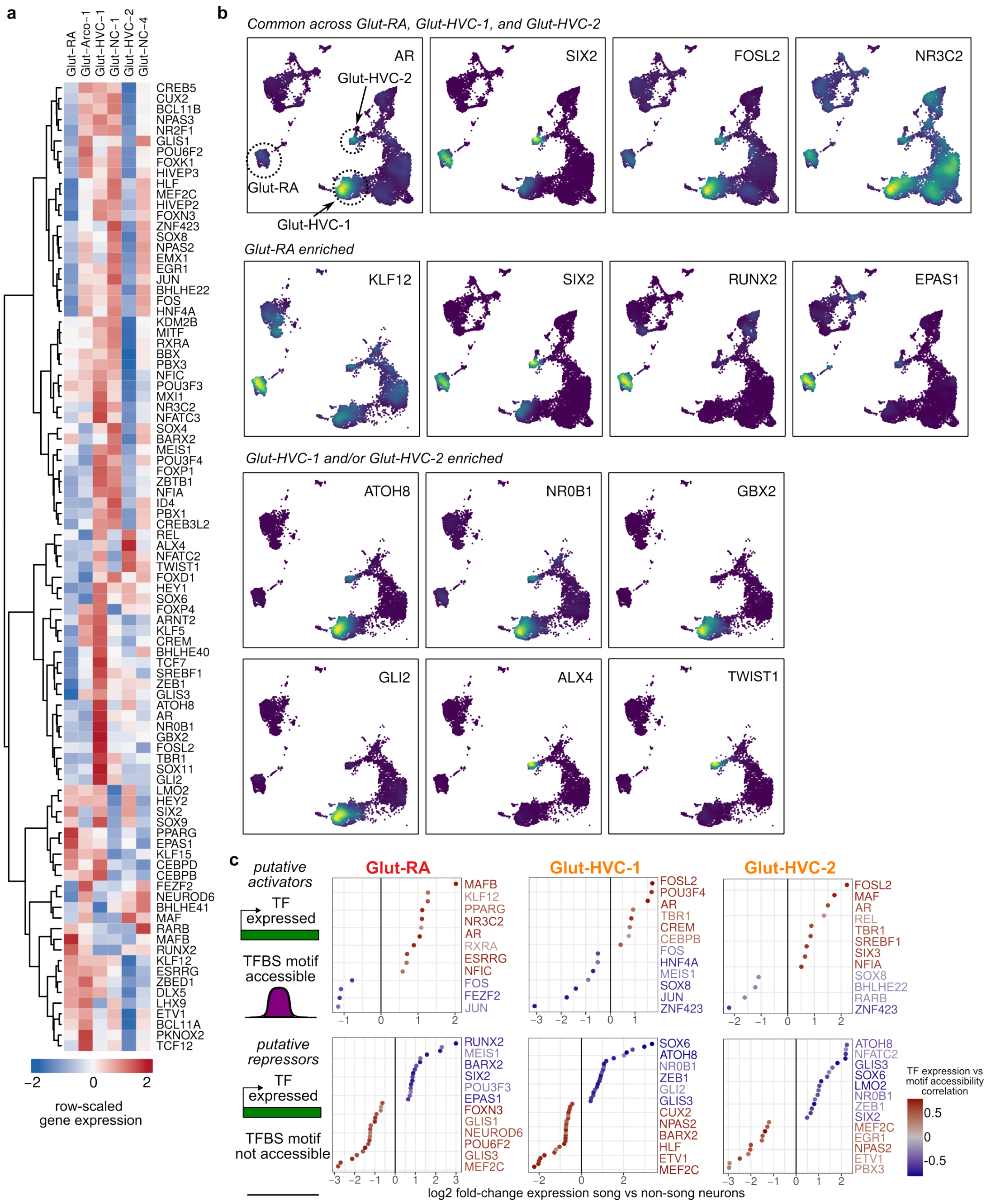

Extended Data Figure 5: Expression of transcription factors differentially expressed across song glutamatergic neurons

(a) Heatmap of transcription factor gene expression in song glutamatergic neurons (Glut-RA, Glut-HVC-1, Glut-HVC-2) and paired non-song neurons (Glut-Arco-1, Glut-NC-1, Glut-NC-4). Expression of each gene is scaled across cell types.
(b) UMAP plots of glutamatergic neurons showing the expression of selected transcription factors between song and non-song glutamatergic neurons.
(c) The accessibility of TFBS motif-containing elements was correlated with the expression of associated transcription factors to identify putative transcriptional activators and repressors that are differentially expressed in song versus non-song glutamatergic neurons. At the right of each plot are transcription factors with the highest or lowest expression different between song and non-song neurons.

bioRxiv preprint doi: https://doi.org/10.64898/2026.04.24.720702; this version posted April 24, 2026. The copyright holder for this preprint (which was not certified by peer review) is the author/funder, who has granted bioRxiv a license to display the preprint in perpetuity. It is made available under aCC-BY 4.0 International license.

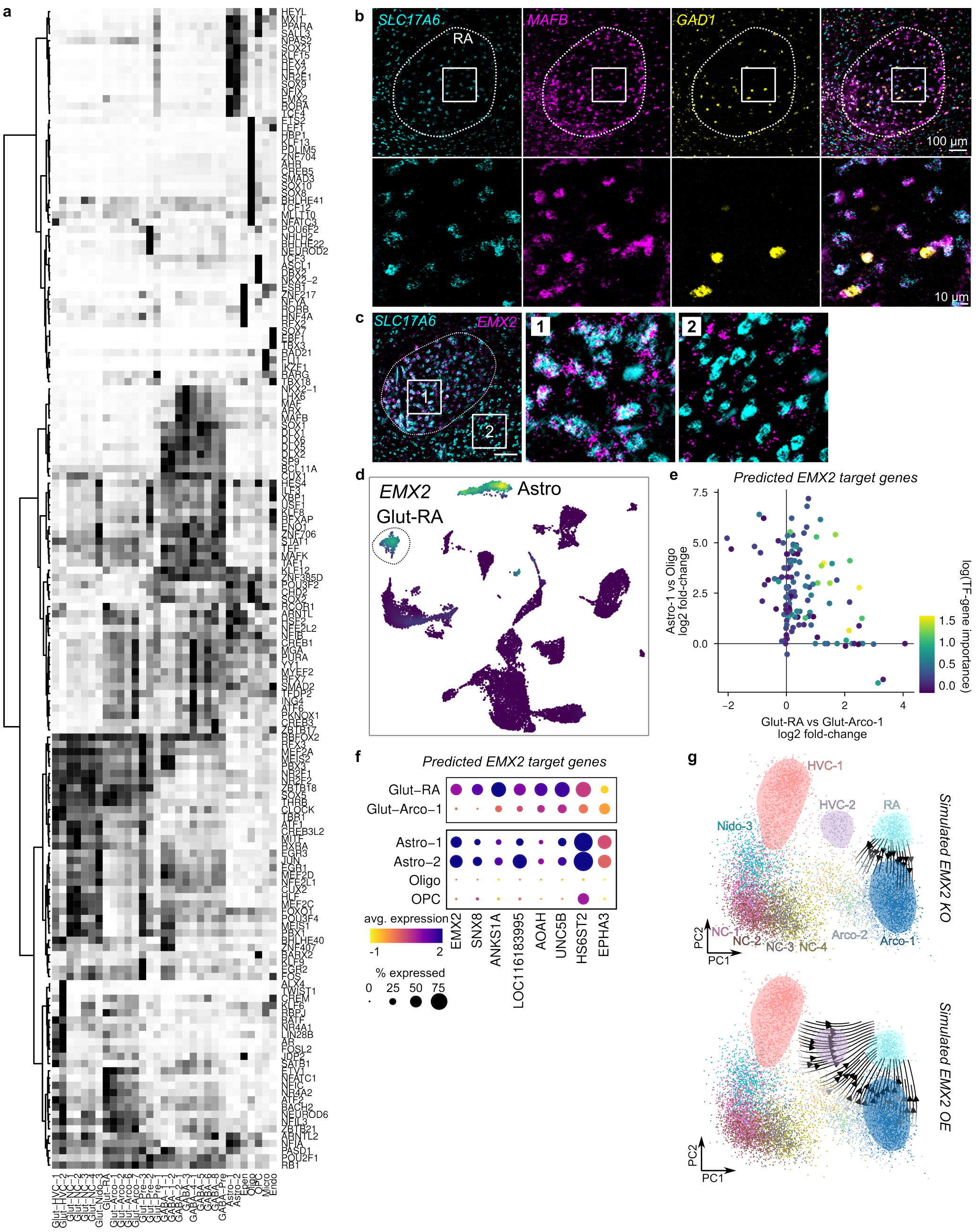

Extended Data Figure 6: Gene regulatory network analysis of song glutamatergic neurons

(a) Expansion of heatmap in Figure 4a.
(b) *In situ* hybridization indicating that MAFB is expressed in glutamatergic (*SLC17A6*-positive) and a subset of GABAergic (*GAD1*-positive) neurons in RA (outlined). However, in motor regions outside of RA that are not involved in song production, *MAFB* is only expressed in GABAergic neurons.
(c) *EMX2* is expressed in glutamatergic neurons in RA but is not expressed in glutamatergic neurons outside of RA. Scale bar is 100 μm.
(d) UMAP plot of the *EMX2* expression. *EMX2* has elevated expression in Glut-RA neurons as well as in astrocytes (*EMX2*).
(e) Differential expression (DE) of predicted *EMX2* target genes between Glut-RA and Glut-Arco-1 neurons compared to astrocytes (Astro-1) and oligodendrocytes (Oligo). Point color indicates TF to target gene importance from SCENIC+ tree-based regression.
(f) Expression of selected genes showing elevated expression in Glut-RA and astrocytes.
(g) Simulated (*top*) knockout and (*bottom*) overexpression of *EMX2* in glutamatergic neurons. Arrows indicate the direction of shifts in neuron position in PCA space following each manipulation.

bioRxiv preprint doi: https://doi.org/10.64898/2026.04.24.720702; this version posted April 24, 2026. The copyright holder for this preprint (which was not certified by peer review) is the author/funder, who has granted bioRxiv a license to display the preprint in perpetuity. It is made available under aCC-BY 4.0 International license.

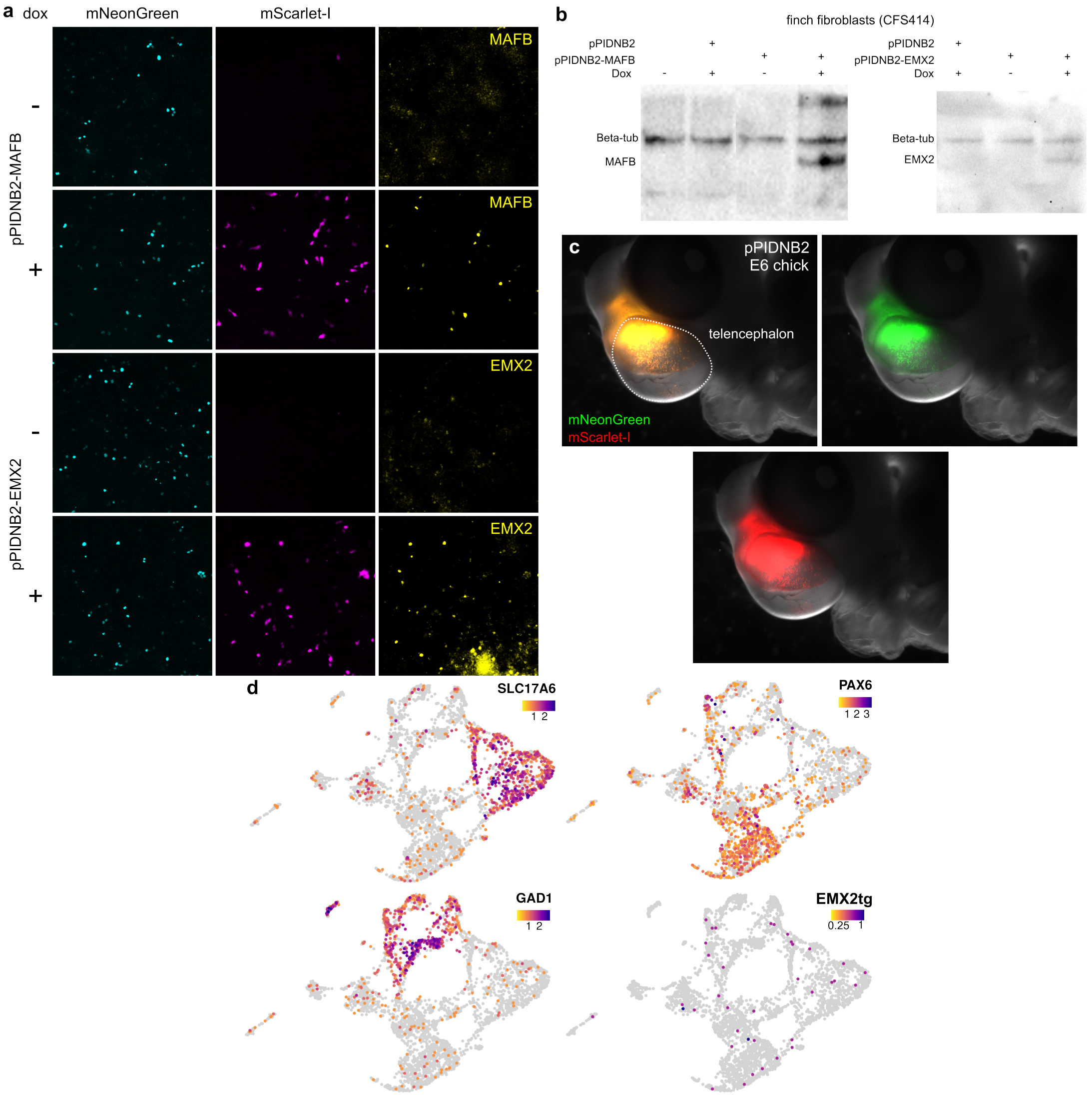

Extended Data Figure 7: Validation of *MAFB* and *EMX2* overexpression constructs

(a) Immunocytochemistry validation of dox-dependent expression of MAFB and EMX2 from pPIDNB2-MAFB and pPIDNB2-EMX2.
(b) Western blot for MAFB and EMX2 overexpression in finch fibroblasts (CFS414) via the pPIDNB2-MAFB and pPIDNB2-EMX2 constructs, using α-MAFB, α-EMX2, and α-β-tubulin antibodies.
(c) Expression of the pPIDNB2 construct in E6 chicken embryos following electroporation at E4 and doxycycline addition at E5.
(d) UMAP plots showing the expression of *SLC17A6* (glutamatergic neurons), *GAD1* (GABAergic neurons), and *PAX6* (progenitor cells).

bioRxiv preprint doi: https://doi.org/10.64898/2026.04.24.720702; this version posted April 24, 2026. The copyright holder for this preprint (which was not certified by peer review) is the author/funder, who has granted bioRxiv a license to display the preprint in perpetuity. It is made available under aCC-BY 4.0 International license.

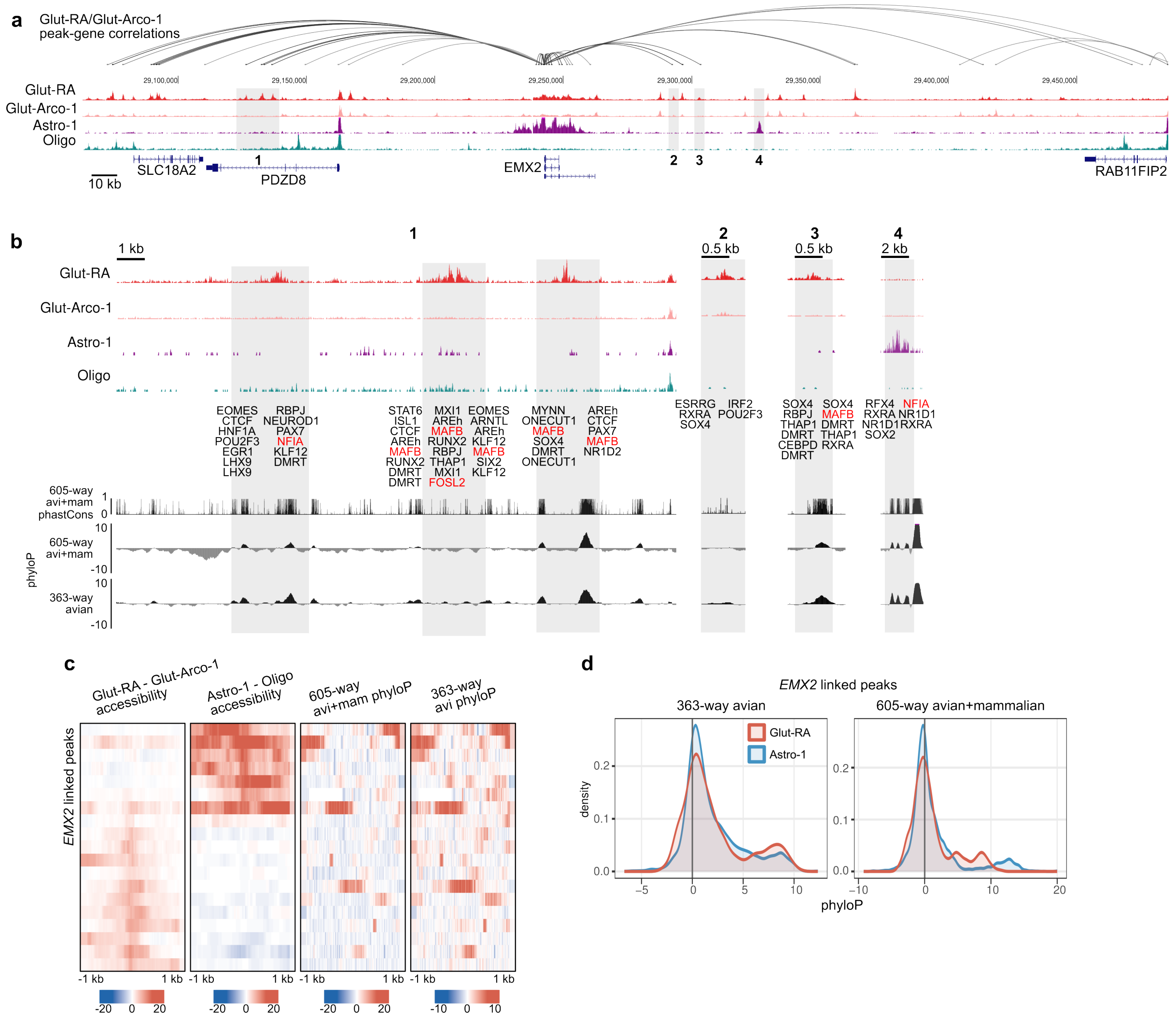

Extended Data Figure 8: Song neuron-specific regulatory landscape of *EMX2*

(a) Chromatin accessibility flanking the *EMX2* locus. Arcs above the accessibility tracks link the MAFB transcription start site with DARs whose accessibilities covary with *EMX2* expression (Pearson r > 0.4).
(b) Magnification of the numbered regions indicated in panel **b**. Transcription factor binding sites for each differentially accessible region are annotated. Sites for SCENIC+ predicted inputs are highlighted in red. AREh, androgen receptor element half-site; AREf, androgen receptor element full-site. At bottom are three tracks showing the conservation (phastCons) or conservation and acceleration (phyloP) across two alignments, one containing 605 species of birds and mammals (avi+mam) and a second with 363 species of birds only.
(c) Differential accessibility and phyloP scores across all DARs linked to *EMX2*.
(d) Distributions of phyloP scores across *EMX2*-linked DARs with high differential accessibility (normalized fragment density differences greater than 5 between Glut-RA and Glut-Arco-1 or Astro-1 and Oligo).
